# PB.43: Review of the determinants of poor screening uptake at City, Sandwell and Walsall Breast Screening Units and the steps taken to improve attendance

**DOI:** 10.1186/bcr3543

**Published:** 2013-11-08

**Authors:** H Khan, S Meraj, A Wilbraham, D Cox, R Bhatt, J Yates, J Waldron, A Powell

**Affiliations:** 1 Sandwell and West Birmingham NHS Trusts, Birmingham, UK; 2 Walsall Manor Hospital, Walsall, UK

## Introduction

The aim of National Health Services Breast Screening Programme (NHSBSP) is to reduce morbidity and mortality related to breast cancer. The success of NHSBSP depends upon a high proportion of women attending for their 3-yearly screening mammograms. The national target for screening attendance is 80% and the minimum standard is 70%. For 2010 and 2011, an overall national screening uptake for women aged 50 to 70 was 73.4%. The overall uptake rate for our screening service is recorded as the fifth lowest in achieving minimum uptake targets. The purpose of this review was to analyse the factors associated with poor uptake in some of our screening regions and assess the effectiveness of the methods used to maximise attendance in these regions.

## Methods

The source of our data collection was west Midlands Cancer Intelligence Unit and NHSBSP database. We analysed the trends in screening uptake from 2007 to 2013.

## Results

Social deprivation, population size, ethnicity, availability and access, and education and awareness are some of the most significant factors that affect uptake. Interventions were devised to target these factors. The initiatives used were simple such as patient letters, promotional posters and multi-lingual leaflets, DNA flyers, telephonic reminders and most importantly collaborative work with GP surgeries. The results were encouraging and we managed to increase the prevalent uptake by 7.87% in our lowest uptake region.
